# Clinical behavior of two-piece zirconia implants. A systematic review

**DOI:** 10.4317/medoral.26752

**Published:** 2025-03-23

**Authors:** Santiago Bazal-Bonelli, María Castro-Janeiro, Julia Ríos-Barbero, Marta Cano Sánchez de Tembleque, Juan López-Quiles, Cristina Meniz-García, Jorge Cortés-Bretón Brinkmann

**Affiliations:** 1 Department of Dental Clinical Specialties, Faculty of Dentistry, Complutense University of Madrid, Spain; 2 Surgical and Implant Therapies in the Oral Cavity Research Group; University Complutense, Madrid, Spain

## Abstract

**Background:**

This systematic review aimed to evaluate the clinical behavior of two-piece zirconia implants (T-PZI) in terms of overall implant survival and success rates, marginal bone loss (MBL) complication rates, and others biological parameters.

**Material and Methods:**

PRISMA guidelines were followed, and the review was registered in the International Prospective Register of Systematic Reviews (PROSPERO). An automated search was conducted in four databases (Medline/PubMed, Scopus, Web of Science, and Cochrane Library), as well as a manual search for relevant clinical articles published until 18 May 2024. The review included human studies with at least five patients in which T-PZI were placed. Quality of evidence was evaluated using the Newcastle-Ottawa Quality Assessment Scale and the Cochrane risk-of-bias tool for randomized trials version 2 (RoB 2).

**Results:**

Six studies met the inclusion criteria and were included for analysis, with a total of 298 T-PZI. A survival rate of 96.31% was recorded with follow-up periods ranging from 18.4±10.4 months to 111.1±2.2 months. The success rate ranged from 63 to 100% and MBL ranged from 0.130.6 to 1.38±0.81mm

**Conclusions:**

T-PZI may offer a reliable alternative to titanium dental implants, achieving a survival rate of 96.31%, accepTable rates of MBL and adequate biological parameters. However, the findings of the review must be treated with caution, as the data obtained are derived from the early stages of this new development in ceramic dental implants. More comparative studies are needed in order to determine the viability of T-PZI in different clinical situations.

** Key words:**Dental implants, zirconium oxide, yttria-stabilized tetragonal zirconium oxide, in-ceram zirconium oxide.

## Introduction

In contemporary implant dentistry, titanium and its alloys are considered the benchmark material for fabricating dental implants (1). But in recent years several questions have arisen about problems that occasionally occur with titanium implants, pointing to a need for an alternative material (2,3). For example, a recent systematic review reports high rates of both mucositis (43%) and periimplantitis (22%) linked to titanium implants (4). Moreover, Wachi *et al*. reported that titanium ions resulting from corrosion may be related to the worsening of mucositis, which subsequently evolves into peri-implantitis leading to bone resorption (5). Another growing issue is titanium hypersensitivity. Several articles have affirmed that some patients may develop clinical signs of allergy to titanium and/or their traces, although this remains uncommon (2,6). Furthermore, titanium implants have a gray color, which might compromise the esthetic results of treatment, especially when placed in anterior areas with a thin gingival biotype (7).

In light of these problems, the use of ceramic materials has been proposed as a possible alternative to titanium (2). Currently, tetragonal zirconia polycrystal, particularly yttrium oxide (yttria) stabilized zirconia is the ceramic of choice for ceramic dental implants (8). In vitro and *in vivo* studies have shown that zirconia implants offer a viable alternative to titanium. The material’s properties include its esthetic white color, good osteointegration and biocompatibility, low bacterial plaque accumulation, low inflammatory infiltrate, good soft tissue integration, and favorable physical properties (9).

**Figure 1:**
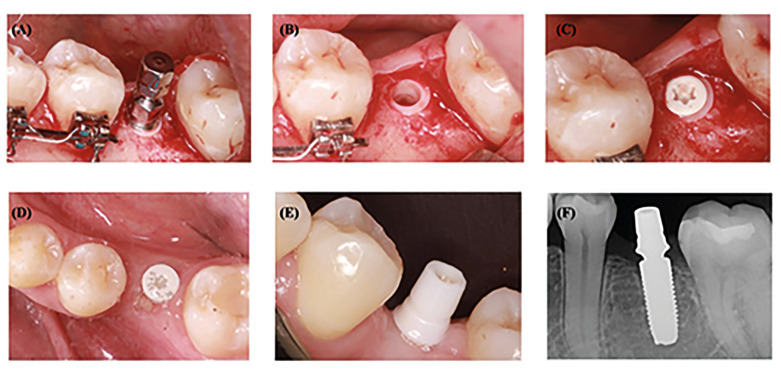
Clinical procedure for T-PZI. (a) implant placement with driver tool. (b) Bone-level implant situation. (c) Implant with cover screw. (d) Implant after the osseointegration period with healing abutment. (e) Implant with definitive abutment (f) Periapical radiograph. Courtesy of Dr. Jorge Cortés-Bretón Brinkmann, Madrid.

At the present time, several ceramic implant brands and designs are available on the market, both one-piece zirconia implants (O-PZI) and more recently T-PZI (Fig. 1). To date, the more widely used and scientifically documented zirconia implant systems are O-PZIs (1). It has been hypothesized that implant manufacturers were initially doubtful that the finer parts of the prosthetic connection in two-piece implants would be able to withstand the loads incurred to the same extent as titanium alloys (10). A recent systematic review (SR) (3) concluded that O-PZI would appear a reliable option for restoring missing teeth, obtaining an implant survival rate of 94.5% and a success rate of 92% after at least 3 years follow-up.

With O-PZI, the absence of a microgap between implant and abutment may seem an advantage, together with the fact that the abutment and abutment-implant junction area is considered the most prone to fracture when unfavorably loaded. But O-PZI presents several drawbacks mainly due to its one-piece presentation, whereby careful case selection and rigorous treatment planning are necessary, as the survival of O-PZI depends on achieving adequate primary stability (3). This means that there is a need to develop two-piece ceramic systems.

Regarding the two options for fixing the abutment (bonding or screw retained), clinical investigations evaluating adhesively bonded implant-abutment interfaces suggest worse outcomes than screw retention. Although screw-retained solutions present sufficient fracture resistance in laboratory testing, investigations of their clinical applicability are still missing (1).

Although several systematic reviews analyzing the clinical performance of zirconia implants have been published recently, all of them analyze one- and two-piece implants together (10-12). However, it must be understood that one-piece and two-piece zirconia implants offer two distinct therapeutic alternatives. It is of the utmost importance for the clinician to be aware that the surgical approaches and postoperative considerations also differ between the two types. For these reasons, a SR involving only T-PZI implants is a necessity if we are to gain a clear overview of the scientific evidence on the subject.

Therefore, the purpose of the present SR was to evaluate the overall survival and success rates, MBL, complication rates, and other biological parameters of T-PZI.

## Material and Methods

- Review development and focused question

This SR followed guidelines established in the PRISMA (Preferred Reporting Items for Systematic Review and Meta-Analyses) statement (13) and was registered in the PROSPERO database (registration number: CRD42023439096). The review was designed with the following PIO(s) (Population, Intervention, Outcome and study design) definitions:

1. Population: Systemically healthy edentulous and partially edentulous patients.

2. Intervention: Two-piece zirconia implant placement.

3. Outcome: Clinical behavior in terms of implant survival, implant success, MBL and biological parameters.

4. Study design: Clinical studies with a minimum sample size of five patients.

So, the review´s PIO(s) question was: In edentulous and partially edentulous patients (P), what is the clinical performance in terms of implant survival, marginal bone loss, implant success and different biological parameters (O) of T-PZI placement (I)?

- Eligibility criteria

Inclusion Criteria:

1. Clinical human studies with a minimum of 5 patients.

2. Minimum follow-up time of one year.

3. Randomized clinical trials (RCTs), prospective cohort studies, retrospective cohort studies, case-control studies or case series.

4. Studies providing the following data: implant survival rate, implant success rate, MBL and other biological parameters.

5. Articles published in English, Spanish or German.

6. Articles published up to 18 May 2024.

Exclusion Criteria:

1. Clinical studies in which O-PZI are placed.

2. Clinical studies in which zirconia implants are placed but do not provide data on their clinical behavior.

3. Cross-sectional studies, animal studies, and case reports.

4. In vitro studies.

- Type of intervention

The review analyzed the clinical performance of the T-PZIs. Studies assessing one or all the following parameters were included: survival rate, MBL, success rates and other biological parameters.

- Data Collection process and data items

The primary objectives of this SR were to analyze implant survival measured as a percentage and MBL measured in millimeters.

Secondary outcomes were success rate measured as a percentage (defined as the absence of mechanical and biological complications during the follow-up period), probing depth (PD) measured in millimeters, bleeding index (BI) (14) measured as a percentage (14), plaque index (PI) measured as a mean score (15) or as a percentage (16), and any intraoperative and postoperative implant complications.

Implant survival was understood as the absence of mobility, without progressive MBL or infection leading to implant removal (17).

- Sources and Search strategy

An automated search was performed in four databases: PubMed/Medline; Web of Science; SCOPUS; and Cochrane Library. The search strategy sought to locate studies published in English, Spanish, and German before May 18th, 2024 using the following search strategy for PubMed/Medline: ("zirconium oxide"[Supplementary Concept] OR (("yttria-stabilized"[All Fields] AND ("tetragon"[All Fields] OR "tetragonal"[All Fields] OR "tetragonality"[All Fields] OR "tetragonally"[All Fields] OR "tetragons"[All Fields])) AND "zirconium oxide"[Supplementary Concept]) OR (("in ceram"[Supplementary Concept] OR "in ceram"[All Fields] OR "in ceram"[All Fields]) AND "zirconium oxide"[Supplementary Concept])) AND ("dental implants"[MeSH Terms] OR "dental implants, single tooth"[MeSH Terms] OR "dental implantation, endosseous"[MeSH Terms] OR "dental implantation"[MeSH Terms]) AND ("two-piece"[All Fields] OR ("two"[All Fields] AND ("piece"[All Fields] OR "pieced"[All Fields] OR "pieces"[All Fields] OR "piecing"[All Fields])) OR ("two"[All Fields] AND "part"[All Fields]) OR "two-phase"[All Fields] OR ("biphase"[All Fields] OR "biphases"[All Fields] OR "biphasic"[All Fields]) OR "bi-phase"[All Fields]). For the other three databases the terms “zirconium oxide”, “dental implants” and “two-pieces” were used in different combinations.

In addition, a manual search was performed by screening the references cited in the articles identified in the electronic search and searching for articles in scientific journals in oral surgery, periodontics and oral implantology that were ranked in the first quartile of the Journal Citation Report (JCR) 2022 and published between April 2023 and April 2024. These journals were: Periodontology 2000, Journal of Clinical Periodontology, Clinical Oral Implants Research, Journal of Periodontology and Clinical Implant Dentistry and Related Research.

To perform the screening process, all references were entered into EndNote X9 Library (Clarivate Analytics, Philadelphia, PE, USA).

- Study selection and screening methods

Two reviewers (S.B.B and M.C.J) made an initial selection of the articles identified in the database and manual searches, screening the titles and abstracts independently. The same reviewers read the full manuscripts of all articles that fulfilled the inclusion criteria detailed above, as well as any papers without sufficient data in the title and abstract on which to base a decision. Any disagreement between the reviewers was resolved by discussion with a third author (J.C.B.B). Inter-reviewer reliability in the selection process and after full text analysis was calculated (percentage of agreement and kappa correlation coefficient). If studies shared the same patient cohort, only the work with the longest follow-up period was selected.

- Risk of bias analysis 

Risk of bias assessment of randomized clinical trials was performed using the Cochrane risk-of-bias tool for randomized trials version 2 (RoB 2). This evaluates five domains: randomization process, deviations from intended interventions, missing outcome data, measurement of the outcome, selection of the reported results, and overall. The results are classified as low risk, high risk or uncertain risk; represented by (+), (-) or (?), respectively (18).

The quality of both prospective and retrospective cohort studies was assessed using the Newcastle-Ottawa Scale (NOS) for cohort studies. The NOS scale evaluates three main perspectives: study group selection, comparability of groups, and outcomes. Studies can score a maximum of 9 points (19). Cohort studies with a single exposure can reach a maximum of 8 points. Studies were classified as good, fair, or poor-quality (GQ, FQ or PQ) following the score algorithm proposed by the Agency for Healthcare Research and Quality (20).

- Data synthesis

Data from each included article was extracted by the reviewers (S.B.B and M.C.J) working together and entered on an Excel spreadsheet (Version 15.17, Microsoft Inc. 2015). In cases of incomplete or missing data, the authors were contacted and asked to supply them if possible. Data were therefore only omitted when they remained unavailable.

The clinical data extracted were as follows: authors, year of publication, study design, number of patients, mean age, mean follow-up duration, number of implants, implant survival, implant success, MBL, PD, BI, PI, and complications.

Meta-analysis could not be performed because of the heterogeneity of the studies reviewed.

## Results

- Study selection

**Figure 2:**
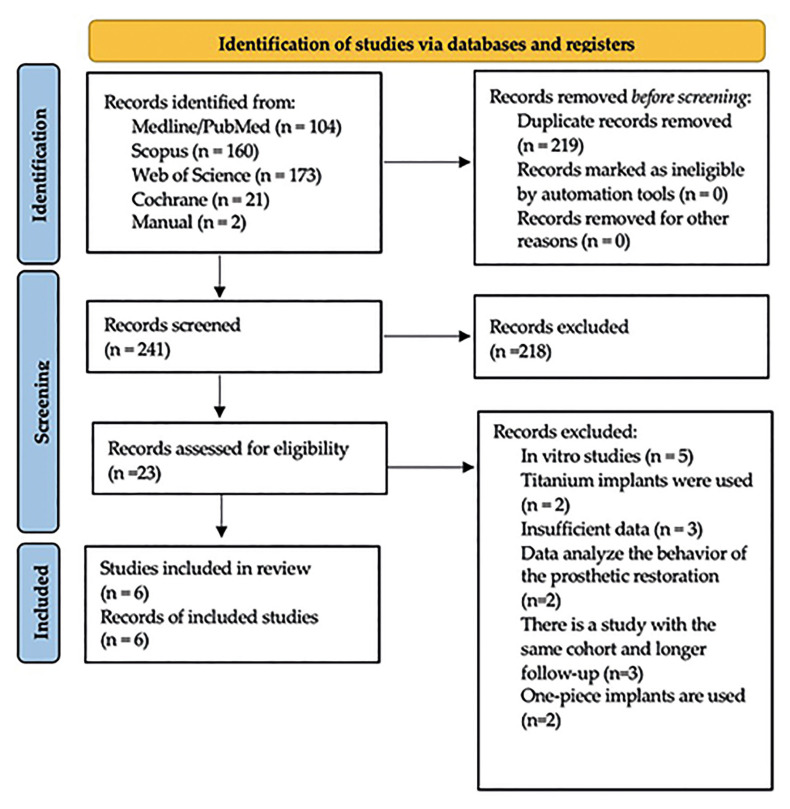
Flow chart illustrates the selection process.

The initial electronic search in databases identified 458 articles and the manual search yielded two additional articles (*n*=460). Of these studies, 219 were duplicates or triplicates and were eliminated. After an initial scan to eliminate articles not relevant to the PIO(s) question, followed by title and abstract screening, a combined total of 23 articles were selected for full-text analysis. Seventeen of these were excluded because they did not meet the inclusion criteria (Supplement 1). Finally, a total of 6 studies were selected for review and data extraction; studies by Brunello *et al*. (21), Cionca *et al*. (22), and Koller *et al*. (23) were continuations of previous studies by the same authors (Becker *et al*. (9), Cionca *et al*. (24) and Payer *et al*. (25), so only the more recent studies with the longest follow-up periods were selected (Fig. 2). shows a flow diagram of the entire article search and selection process. Meta-analysis could not be performed due to the heterogeneity of the studies.

- Study characteristics

Among the six included articles, four of them were prospective cohort studies (21,22,26,27); one was a randomized clinical trial (RCT) (23); and one was a retrospective cohort study (28). Table 1 and Table 2 summarize basic information extracted from the articles, including the number of patients, number of implants placed, implant position and brand, implant survival, implant success, prosthesis type, biological parameters, follow-up periods, and complications.

- Risk of Bias and Quality Assessment

The NOS scale was used to assess the quality of the four prospective cohort studies (21,22,26,27) and the one retrospective cohort study reviewed (28). The prospective studies scored 7 (low bias) (26) and 6 points (medium bias) (21,22,27) and the retrospective study scored 6 points (medium bias) (28). These scores point to an adequate quality of evidence among these studies (Table 3).

The single RCT (23) was assessed with the Cochrane risk-of-bias tool (RoB 2). The randomization process, deviations from intended interventions, and the selection of reported results were all adequate, so the risk of bias was low (Table 4).

- Synthesis of results

Inter-review agreement: Cohen’s Kappa statistic between the two reviewers (S.B.B and M.C.J) was 0.847 (CI 95% 0.884-0.809) for title and abstract selection and 0.905 (CI 95%: 0.935-0.875) for full text assessment. Therefore, the level of agreement was considered strong agreement and almost perfect respectively. In any case, minor discrepancies were assessed jointly between the two reviewers; agreement was reached without recourse to the consensual assessment of the third reviewer.

Patient characteristics: The six studies provided a total of 298 T-PZI; the follow-up periods ranged from 15.0±2.1 months to 111.1±2.2 months. The exact number of patients could not be established because in one study (28) the authors did not report this datum clearly, nevertheless the mean age of the patients could be extracted, being 49±12.8 years to 54.0±8.6 years. Table 1 shows all the characteristics of patients, and the implants placed.

Implant survival rate: All included studies evaluated the survival rates of TPZIs. The survival rate of the TPZIs ranged from 83% to 100%. The number of implants that survived during the follow-up periods was 287, therefore the weighted mean survival rate was 96.31%.

Success rate and marginal bone loss: Regarding implant success, only two of six studies (22,27) reported the success rate (63% and 100% respectively). One of the studies (22) followed the success criteria of Albrektsson *et al*. (17) and the other study (27) took as reference the success criteria of Buser *et al*. (29) and Kohal *et al*. (30). In addition, Cionca *et al*. (22) calculated cumulative rates after 6 years using the Kaplan-Meier method.

Three (22,23,28) of the five studies reported MBL, which ranged from 0.13±0.6 to 1.38±0.81mm.

Cumulative rates were calculated after 1 and 6 years using the Kaplan-Meier method.

Biological parameters: PD, BI and PI

Five of six studies included data about biological parameters (21-23,26,28). PD was expressed as a mean score measured in millimeters (mm) and analyzed in four studies (21,22,26,28), with values ranging from 1.80±0.40mm to 3.5±1.0mm. Regarding BI, this was defined as bleeding on probing (BoP) expressed as a percentage (%). The data ranged from 4.1±4.2% to 50.0% and were reported in all five studies (21-23,26,28). In turn, PI was defined either as Plaque Index, in two studies (22,23), or modified PI according to Mombelli (15), in two other studies (21,26). The difference between the two is that the former is expressed as a percentage and the latter as a mean score. Due to the scarcity of data and their heterogeneity, meta-analysis of biological parameters could not be performed. However, the results obtained for PI ranged from 11.07%±8.11% to 31%, while the results for modified PI ranged from 0.38±0.68.

Complications: Regarding complications, one of the studies included in the review did not report any type of complication (26), two studies did not describe whether or not complications occurred (23,28), and the remaining three studies described 32 biological complications and 19 mechanical or prosthetic complications (21,22,27). The complications recorded are detailed in Table 2.

## Discussion

The aim of this SR was to analyze the clinical behavior of T-PZIs, evaluating survival and success rates, as well as MBL and biological parameters PD, BI, and PI. A total of 6 clinical studies (5 cohort studies and one RCT) with 298 implants were included for review.

A mean survival rate of 96.31% was recorded with follow-up periods ranging from 15.0±2.1 months to 111.1±2.2 months. This result is comparable with that established in a recent SR focusing exclusively on O-PZIs, which showed a survival rate after a 3-year follow-up of 94.5% (95% CI 90.4 - 98.6%, *p*<0.001) (3). It should be noted, however, that the follow-up times in the studies reviewed here were more heterogeneous and that fewer implants were analyzed (298 vs. 1621).

It also should be considered that to date, most of the available and scientifically documented zirconia implant systems have been O-PZI.

The advantages of the O-PZI lie in the absence of a microgap between implant and abutment. In principle, this should minimize bacterial infiltration and reduce the risk of fracture between the abutment and the implant - the area most prone to fracture (2,31). In turn, the prosthodontic restoration resembles the behavior of the natural tooth, which makes it easier to take impressions, as prosthetic abutments may be dispensed with (31). However, the O-PZI suffers several drawbacks. The placement of these implants requires experienced surgeons and prosthodontists due to its restricted flexibility in cases of compromised angulation or vertical positioning (1). In turn, angled abutments to correct any misalignment remain unavailable (2). Cementation is the only option for connecting prosthodontic elements. So, if there is a problem with the restoration, retrieval will be more complicated (3). If cement has not been managed with due care, there is an increased biological risk in the periodontal area due to cement extravasation (32).

In addition, the survival of O-PZI depends on achieving sufficient primary stability. O-PZI also require a load-free healing period, a problem given that the supra-mucosal part of the implant will be subjected to the forces of mastication and tongue movement immediately (3,15). For this reason, the patient’s collaboration and careful post-surgical monitoring are crucial to a successful outcome.

Faced with this scenario and the inherent limitations of O-PZI, two-piece systems have been developed. Their advantages include the possibility of protected osseointegration beneath the soft tissue; this reduces surgical difficulty as primary implant stability is not mandatory and offers a better possibility of simultaneous bone augmentation (33). Two-piece systems permit the use of angled abutments to correct possible misalignments, and the crowns can be screw-retained, reducing the biological risk of cement extravasation (31).

Nevertheless medium/long-term clinical investigations of T-PZI have been scarce. Further research is necessary, especially given that T-PZI has two parts, making the prosthetic connections with very thin structures in some areas, which might make it more susceptible to fracture (34). Regarding this possibility, a European Association for Osseointegration (EAO) position paper on bonded implant-abutment interfaces suggests that T-PZI may present inferior outcomes. At the same time, the feasibility of screw-retained solutions has only been sufficiently demonstrated in laboratory research (1). Only one of the six articles included in the present SR (with 24 implants) used screw retention, with a survival rate of 100% after a follow-up of 15.0±2.1 months (26).

Although the survival results of T-PZI implants in the present SR are promising, they are inferior to those observed for titanium implants. In this respect, Jung *et al*. (35) established survival rates of 97.2% at 5 years and 95.2% at 10 years for titanium implants supporting single crowns (SCs), while Pjetursson *et al*. (36) obtained survival rates of 97.2% after 5 years and 93.1% after 10 years for implants supporting fixed dental prosthesis (FDPs). Nevertheless, the small number of T-PZI implants investigated to date and the shorter follow-up periods involved make comparison difficult at this stage.

Regarding the success rate of T-PZI implants, only two articles provided this information (22,27). These authors observed success rates of 63 % and 100 % respectively. However, the disparity and paucity of clinical results in this regard make any comparison with O-PZI and titanium implants unfeasible.

Three of the studies reviewed reported MBL (22,23,28). Marginal bone loss is understood to be a significant parameter for assessing the success of implant-based rehabilitations. A vertical MBL around implants of up to 1.5mm to 2mm is regarded as accepTable during the first year of functional loading (37). Thereafter, annual loss of 0.2mm is thought permissible (17). The three studies that reported these data did fulfill these success criteria, with MBL ranging from 0.13±0.6 to 1.38±0.81mm (22,23,28).

With respect to probing depth and biological parameters (PI and BoP), it may be affirmed that the implants met the criteria for peri-implant health. However, the high percentage of BoP in the study by Lorenz *et al*. (26) is noTable (50% of the implants included in the study). The authors observed a statistically significant increase between baseline (T0) and measurement after six months’ loading. Nevertheless, these authors explain that in spite of 50% of the implants presenting BoP - a potential expression of peri-implant mucositis - no patients were diagnosed with peri-implantitis (presence of BoP and PPD ≥ 6 mm).

With regard to complications observed in the studies analyzed, the high number of bonded abutment fractures in the studies by Cionca *et al*. (22) and Brunello *et al*. (21) are noTable, together with the clinical difficulty involved in the retrieval of these implants after fracture. In fact, the ceramic implant design used in the study by Cionca *et al*. (22) (Zeramex® T, Dentalpoint AG) was withdrawn from the market in 2013 and is no longer commercially available. These data contrast with the absence of complications associated with T-ZPI with screw-retained abutment in the single study included (26).

Given the drawbacks of O-PZIs, it seems logical that clinical research should focus on the medium/long-term results of T-PZIs with screw-retained abutments, since the admittedly scant research conducted to date suggests that screw retention has a positive impact on T-PZI performance.

The present SR suffered some limitations, particularly the heterogeneity of the studies analyzed and the scarcity of randomized controlled clinical trials comparing titanium implants with T-PZI. The total number of implants included was small and there were large differences in the follow-up times between studies reviewed. Moreover, different ways of fixing the abutment were used and some of the prototype implants described in the studies are no longer commercially available. Studies with longer follow-up periods are needed to confirm the present findings, as well as to investigate all the variables that could influence outcomes.

## Conclusions

Despite this systemic review’s limitations, T-PZI may offer a reliable alternative to titanium dental implants, achieving a survival rate of 96.31% over follow-up periods ranging from 15.0±2.1 months to 111.1±2.2 months. Nevertheless, the findings of the review must be treated with caution, as the data obtained are derived from the early stages of this new development in ceramic dental implants. The results point to accepTable rates of MBL and adequate biological parameters. More comparative studies are needed - well-designed randomized clinical trials with sufficient sample sizes and follow-up periods - in order to determine the viability of T-PZI in different clinical situations.

## Figures and Tables

**Table 1:** Information about selected studies including study design, number of patients, gender, mean age, number, location and type of implants, prothesis type and follow-up.

Author and year	Study design	Patients (number)	Gender (male/female)		Mean age (years)	Implants (number)	Implants (location)	Implants (type)	Prosthesis (type)	Mean follow-up (months)
Brunello *et al.* 2022 (21)	Prospective cohort	60	-	-	49±12.8	52	-	ZV3, Zircon Vision GmbH	SCC	111.1±2.2
Cionca *et al.* 2021 (22)	Prospective cohort	32	14	18	51.9	49	Maxilla: 24 Mandible: 25	Zeramex® T	SCC	82.2±5.86
Koller *et al.* 2020 (23)	RCT	11	-	-	46	16	Maxilla: 3 Mandible: 13	Ziterion®	SCC	80
Lorenz *et al.* 2022 (26)	Prospective cohort	19	13	6	54.0±8.6	24	Maxilla: 24	Pure ceramic implant Straumann®	SSC	15.0±2.1
Brüll *et al.* 2014 (28)	Retrospective cohort	-	-	-	51	66	-	-	SSC and MCC	18.4±10.4
Karapataki *et al.* 2023 (27)	Prospective cohort	39	17	22	58.5	91	-	Patent Zircon Medical Management	SSC and MCC	74.6±21.4

RCT: Randomized clinical trial; SCC: single cemented crown; SSC: S screwed crown; MCC: multiple cemented crowns.

**Table 2:** Information about selected studies including implant survival rate, implant success rate, marginal bone loss, probing depth, bleeding index, plaque index and complications.

Author and year	Implants (number)	Survival rate (%)	Success rate (%)	MBL (mm)	PD (mm)	BI (%)	PI	Complications
Brunello *et al.* 2022 (21)	52	96	-	-	3.0±0.6	12.9±15.8 (BoP)	0.33±0.28	10 mucositis 10 peri-implantitis 6 fracture fiberglass abutments
Cionca *et al.* 2021 (22)	49	83	63	0.05±0.85	3.5±1.0	28.5 (BoP)	31%	3 peri-implantitis 1 implant fracture 6 abutments fracture 6 loss retention in crown- abutment complexes
Koller *et al.* 2020 (23)	16	87.5	-	1.38±0.81	-	16.43±6.16 (BoP)	11.07%±8.11%	-
Lorenz *et al.* 2022 (26)	24	100	-	-	2.49±0.49	50.0% (BoP)	0.38±0.68	No complications
Brüll *et al.* 2014 (28)	66	97	-	0.13±0.6	1.80±0.40	4.1±4.2 (BoP)	-	-
Karapataki *et al.* 2023 (27)	91	100	100	-	-	-	-	9 mucositis

MBL: Marginal bone loss; PD: Probing depth; BI: Bleeding index; PI: Plaque index; BoP: Bleeding on probing.

**Table 3:** Quality assessment of cohort studies using the Newcastle-Ottawa Scale.

Study	Selection				Comparability		Outcome			Number of stars (out of 9)
	S1	S2	S3	S4	C1	C2	E1	E2	E3	
Brunello *et al.*2022 (21)	★	★	★	★	0	0	0	★	★	6
Cionca *et al.*2021 (22)	★	★	★	★	0	0	0	★	★	6
Lorenz *et al.*2022 (26)	★	★	★	★	0	0	★	★	★	7
Brüll *et al.*2014 (28)	★	★	★	★	0	0	0	★	★	6
Karapataki *et al.*2023 (27)	★	★	★	★	0	0	0	★	★	6

★=1.

**Table 4:** Quality assessment of included studies using the Cochrane risk-of-bias tool for randomized trials version 2 (RoB 2).

Study	Randomization process	Deviations from intended interventions	Missing outcome data	Measurement of the outcome	Selection of the reported results	Overall
Koller *et al.* 2020 (23)	+	+	?	?	+	+
